# The relationship between childhood maltreatment and mental health problems: coping strategies and social support act as mediators

**DOI:** 10.1186/s12888-022-04001-2

**Published:** 2022-05-27

**Authors:** Yingying Su, Xiangfei Meng, Guang Yang, Carl D’Arcy

**Affiliations:** 1 Department of Psychiatry, Faculty of Medicine and Health Sciences, McGill University and the Douglas Research Centre, Montreal, QC Canada; 2 School of Public Health, University of Saskatchewan, Saskatoon, SK Canada; 3 Department of Psychiatry, College of Medicine, University of Saskatchewan, 103 Hospital Drive, Saskatoon, SK S7N 0W8 Canada

**Keywords:** Child maltreatment, Mental health, Social support, Psychological adaptation, Structural equation modeling

## Abstract

**Background:**

Childhood maltreatment significantly increases the risk of developing mental health problems in adolescence and adulthood. The present study examines if coping strategies and social support mediate the relationship between childhood maltreatment and mental health problems.

**Methods:**

Data analyzed were from the 2012 Canadian Community Health Survey Mental Health (CCHS-MH, *N* = 25,113), a national population survey. A structured diagnostic interview, the World Health Organization version of Composite International Diagnostic Interview (WHO-CIDI), was used to assess mental health status. Multiple mediation analysis with structural equation modelling is used to test the mediating effects of coping skills and social support in the relationship between childhood maltreatment and mental health problems.

**Results:**

Our findings demonstrate that both coping strategies and social support mediated the link between childhood maltreatment and major depressive episode (mediation proportion: 18.3%), generalized anxiety disorder (mediation proportion: 19.8%), and suicide ideation (mediation proportion: 15.9%). By and large, the study results showed that coping skills and social support had both direct and indirect effects on the studied mental health problems with coping skills having a stronger impact.

**Conclusions:**

Personal resources play an important resilience role in the associations between maltreatment and mental disorders with positive coping strategies, an internal resource, having a stronger protective presence. This research reinforces the need for strengthening positive coping strategies as well as social support as preventive strategies to improve mental health for individuals who have experienced childhood maltreatment.

**Supplementary Information:**

The online version contains supplementary material available at 10.1186/s12888-022-04001-2.

## Introduction

Childhood maltreatment refers to any act of commission or omission by parents or other caregivers resulting in potential threat of harm to a child [1]. It is a significant public health concern with a considerable global prevalence [2–4]. Maltreated individuals have more cognitive, emotional, and behavioral difficulties [5, 6]. In addition, a history of child maltreatment predicts an increased risk of subsequent mental health problems, such as depression, anxiety, alcohol abuse, and suicide thoughts [7–9]. However, not all who have experienced childhood maltreatment go on to develop negative mental health consequences. Some individuals are or become more resilient than others [10].

Resilience is a protective factor in the face of significant childhood adversity [11], it can minimize the negative impact of the adversity on subsequent well-being [12]. Building on the Bronfenbrenner’s ecological model [13], individuals encounter different environments throughout the lifespan that may influence behaviors in varying degrees. Their unique reactions and self-regulatory processes following maltreatment serve as important intervening factors through which child maltreatment influences mental health status. Two well-studied personal resources, coping strategies (internal) and social support (external), have been found to be involved in the pathway from childhood maltreatment to subsequent adaption and mental health well-being [14].

Coping strategies are defined as dynamic changes in cognitive and behavior efforts to manage or reduce the internal and/or external demands that are created by stressful events [15]. Schulte et al. demonstrated that children exposed to maltreatment might lack the necessary experiences to develop positive problem-focused coping skills that usually emerge during childhood and develop rapidly during later childhood and into early adolescence [16]. Likewise, Arslan et al. in a cross-sectional study found that childhood maltreatment may influence the development of youths’ adaptive coping strategies that in turn have been found to be related to youths’ better mental health and well-being [17, 18]. Carver et al. observed a negative correlation between psychological and physical maltreatment and emotion-focused coping strategies [19]. In addition, evidence has documented the temporal association between coping strategies and mental health problems. For instance, Richardson et al. observed that avoidant coping predicted subsequent psychopathology, for example, generalized anxiety disorder, and certain pathology [20]. Similarly, Kim et al. in a longitudinal cohort study involving twenty-one biweekly waves of assessment found that positive coping strategies enhance mental health and well-being and were predictive of a reduction in depressive symptoms [21].

It is reasonable to postulate that different coping strategies could mediate the relationship between maltreatment and subsequent mental well-being. The importance of coping as a mediator between childhood maltreatment and its responses has been theorized in most models of stress and coping. The theoretical perspectives on stress and coping further suggest that the severity of mental disorders is shaped by coping strategies that arise as a direct consequence of experiencing stress [22]. Mengo et al. in their cross-sectional study found that coping strategies mediated the relationship between abuse and mental health issues [23]. A recent study also confirmed that coping strategies mediated the relationship between childhood maltreatment and suicidal ideation [24].

Social support refers to informational, emotional, or tangible support from others [25]. It promotes better physical health, cognitive functioning and psychological health [26, 27]. For children and youth, social support plays a crucial role for their normal development and mainly comes from family, peers, teachers and caregiving relationships [28]. The social support deterioration model conceptualizes that stress erodes the perceived availability or effectiveness of social support, and then leads to various short- and long-term effects on mental health issues [29]. Social support can serve as a mediator and explain some of the effects of childhood maltreatment on mental health outcomes. The fact that stress leads to corrosion of social connections and ensuing challenges dealing with adversity, which in turn precipitates mental health issues [30]. For those with exposures to child maltreatment, lack of social support means the absence of positive mentoring from their adult role models which limits their opportunities to develop competencies and skills conductive to better mental health in the later life [31]. Sperry and Widom addressed this issue in their prospective cohort study involving adults with a history of childhood maltreatment [32]. They found that maltreated individuals reported lower levels of perceived social support in adulthood compared to those who never had the exposure to maltreatment. Furthermore, social support has been found to mediate the association between childhood maltreatment and anxiety and depression in adulthood [33]. Evidence increasingly supports the mediating role of social support in the relationship of child maltreatment and adult mental health problems (such as feeling depressed or anxious) [31]. Lagdon et al. in a cross-sectional study found that social support played a mediating role in the relationship between childhood maltreatment and mental disorders including depression and anxiety [34]. Similarly, Struck et al. in an ongoing multicenter study also found that the effect of emotional abuse and emotional neglect on depression is mediated by social support [35]. In summary, stress, coping, as well as social support theory suggest that both coping strategies and social support are involved in the relationship between child maltreatment and mental health outcomes [23]. In line with the resilience theory, social support and coping could protect individuals with childhood maltreatment from mental disorders [36]. However, previous prospective cohort studies were conducted in selected study populations that were mostly composed of adolescents, older population, and patients with mental disorders. Thus, they had relatively narrow generalizability. We were not aware any published studies that examined the effect of social support and coping strategies in the association between childhood maltreatment and common mental illness in a nationally representative sample (or in a Canadian setting). The present study aims to examine the effect of social support and coping strategies in mediating the association between childhood maltreatment and mental disorders in a large national epidemiological survey of mental health in the Canadian population (*N* = 25,113). The present study extends the current literature by simultaneously considering the buffering effect of social support *and* coping strategies in the stress process in an integrated structural model. We also expand the range of psychiatric outcomes studied. We hypothesized that: 1) social support and coping strategies mediate the associations between childhood maltreatment and specific mental disorders – major depressive episode (MDE), generalized anxiety disorder (GAD), and suicide ideation later in life; and 2) the buffering effects of coping skills and social support contribute differently to these associations.

## Methods

### Data and sample

Data analyzed were from the Public Use Microdata file (PUMF) of the 2012 Canadian Community Health Survey-Mental Health (CCHS-MH 2012), which was a national cross-sectional population-based survey conducted by Statistics Canada between January 2, 2012, and December 31, 2012. CCHS-MH 2012 is still the most recent survey devoted to exploring on the mental health status and behaviors of Canadians. The CCHS-MH 2012 targeted household residents 15 years of age and older living in any of the 10 provinces and 3 territories of Canada. Sampling *excluded* those living on First Nations reserves and other Aboriginal settlements, full-time members of the Canadian Forces and the Royal Canadian Mounted Police (RCMP) as well as the institutionalized population. It is estimated these exclusions account for 3% of the national population. A multistage stratified cluster design based on the monthly national Labor Force Survey was used to ensure adequate coverage by sex and age group for each province and territory. Survey respondents were interviewed using computer assisted personal interviewing (CAPI) and telephone interviewing (CATI) technology. Participation in the survey was voluntary. The interviews were conducted in either French or English, Canada’s official languages. The vast majority of interviews (87.0%) were conducted in person at participant’s home. A total of 25,113 participants completed the survey. The combined individual- and household-level response rate was 68.9% [37].

Participants in the original survey signed an informed consent and voluntarily participated in the survey. The original survey received ethical approval through Statistics Canada procedures. The CCHS-MH PUMF is an anonymized survey data file devoid of any personally identifying information. The secondary analysis of such data does not require ethical clearance.

### Measures

#### Child maltreatment

Respondents were asked about the frequency of experiencing physical abuse, sexual abuse and exposure to intimate partner violence prior to the age of 16. The exposure to intimate partner violence, and physical abuse was assessed using items from Childhood Experiences of Violence Questionnaire (CEVQ), a brief 18-item self-report measure. CEVQ has been shown to be reliable and valid in assessing maltreatment among youth in non-clinical settings [38]. Three questions were used to evaluate physical abuse, assessing the number of times respondents experienced being: 1) slapped, hit or spanked by an adult; 2) pushed, grabbed, shoved or thrown at by an adult; or 3) physically attacked (kicked/bitten/punched/choked/burned/ other) by an adult. One item was used to assess exposure to intimate partner violence, it asked how many times the respondent saw or heard caregiver hit another adult. The two questions evaluating how frequently sexual abuse occurred were: did the respondent: 1) experienced forced or attempted forced sexual activity; or 2) experienced sexual touching. Respondents were asked about the frequency of childhood abuse occurrence using a five-category ordinal scale: never, 1–2 times, 3–5 times, 6–10 times, or > 10 times. The sexual abuse items were very similar to the ones used in previous Statistics Canada population-based surveys [37]. The Cronbach alpha for the childhood abuse items was 0.79.

#### Social support

The current social support was assessed with Social Provisions Scale-10 item (SPS-10). It is a shortened version of the 24-item Social Provisions Scale developed by Cutrona and Russell [39]. It was originally designed to measure six provisions of social support on a scale from 1 (“strongly agree”) to 4 (“strongly disagree”): emotional support or attachment, social integration, reassurance of worth, material help, orientation, and opportunity for nurturance. The short form of the scale deleted the opportunity for nurturance domain. The SPS-10 has been validated in a French-speaking population by Caron [22]. The Cronbach’s alpha for the SPS-10 in this study was 0.92.

#### Positive coping skills

Current positive coping skills were assessed using the following two items, which were used in a previous study and in the CCHS-MH 2012 [40]. Respondents were asked about their: 1) self-perceived rating of ability to handle unexpected and difficult problems; and 2) self-perceived rating of ability to handle day-to-day demands. The response categories were a 5-level Likert scale ranging from poor to excellent (1 = poor, 5 = excellent). Its Cronbach alpha’s value was 0.72.

#### Common mental health disorders

The World Health Organization version of Composite International Diagnostic Interview (WHO-CIDI) structured diagnostic interview was used to assess the presence of major depressive episode and generalized anxiety disorder during the past 12 months prior to the diagnostic interview [40]. The WHO-CIDI is a standardized measure based on the Diagnostics and Statistics Manual of Mental Disorder-4th edition (DSM-IV) and International Statistical Classification of Diseases and Related Health Problems-10th revision (ICD-10) [41, 42]. Suicide ideation was assessed by self-reports of suicidal thoughts in past 12 months. Respondents who answered yes to the presence of such thoughts were coded as 1 (positive); otherwise, they were coded as 0 (negative).

### Statistical analysis

Zero-order and partial correlations between each variable were calculated. Structural Equation Modeling (SEM) was performed to test the hypothesized effects of social support and positive coping strategies in the relationships between childhood maltreatment and three mental health problems. First, Baron and Kenny’s 4-step approach was used as the strategy for testing the mediating effect, including: 1) the independent variable has a direct effect on the mediator; 2) the independent variable has a direct effect on the dependent variable; 3) the mediator has a direct effect on the dependent variable; and 4) after the mediator is entered in the regression model, the relationship between the independent and dependent variables either disappears (full mediation) or is significantly diminished [43]. Next, structural Equation Modeling (SEM) was used to test the hypothesized effects of social support and positive coping strategies in the relationships between childhood maltreatment and three mental health problems. Confirmatory factor analyses (CFA) were performed to test the sufficiency for the factorial validity and reliability of the measured associations among the latent variables [44]. Diagonally weighted least squares mean and variance adjusted test statistic (WLSMV) were applied in this study. This is a robust approach providing the best option for modelling categorical or ordered variables that does not assume that the variables are normally distributed. The total effect of childhood maltreatment on mental health outcomes is the sum of direct and indirect effects. The proportion of the effect of childhood maltreatment on mental health related outcomes mediated by the mediators is calculated as the indirect effect divided by the total effect. The mediation proportion (MP) is given by: MP = $\frac{Indirect\ effect}{Direct\ effect+ Indirect\ effect}$. Considering the study’s sample size the root mean square error of approximation (RMSEA) and the Comparative Fit Index (CFI) were used as indicators of goodness of fit. The RMSEA, is a parsimony-adjusted index, which can take model complexity into account, it is argued to be the least affected by sample size [45]. The CFI, an incremental fit index, estimates differences between the examined model and a null model [46]. Hu and Bentler suggested that models presenting RMSEA below 0.06 and CFI indices above 0.95 are generally considered to be acceptable [47]. Finally, to assess the significance of the mediation effects, bias corrected method of model-based bootstrap sampling with 1000 iterations was used to estimate indirect effects and confidence intervals (CIs).

Missing values were imputed using a multiple imputation procedure as suggested by Sterne et al. [48] with STATA. We created five imputed datasets using the fully conditional specification method in STATA- the multiple imputation procedure. These five imputed datasets were used as parallel samples for conducting SEM. STATA command ‘Midiagplots’ was used to compare the distributions of the imputed data and the observed data to ensure the quality of the predictions of the imputation models [49]. Statistical significance for all data analyses was set at *p* < 0.05 (by two-tailed test). Analyses on the multivariate relationships among studied variables were performed with R Studio [50] with R 3.3.1 [51]. The structural equation model was tested using the R package Lavaan [52].

## Results

### Characteristics of the study sample

A total of 25,113 individuals were included in the present study. Socio-demographic and lifestyle characteristics of the study samples are summarized in the Table 1. The missing data on these variables ranged from 0.03 to 0.57% (Race/ethnicity: 0.44%; Marital status: 0.27%; Household income: 0.06%; Immigration status: 0.57%; Type of smoker: 0.06%; Type of drinker: 0.13%; Have chronic conditions: 0.11% and Perceived health: 0.03%). In general, the majority of respondents were females (54.8%), white (83.5%), non-immigrants (83.1%), married/or in a common-law relationship (50.3%), with a high level of education (56.1%), and $80,000 CAD was the most frequently reported income category, it was reported by 37.8% of the respondents. More than half (52.9%) of the study samples had at least one type of childhood maltreatment. Among maltreated victims, majority of individuals (73.7%) experienced one to two types of childhood maltreatment.

**Table 1:** Sociodemographic and lifestyle characteristics of the study sample (*N* = 25,113)

Variables	Frequency	%
Age (years)		
15–24	4013	16.0
25–44	6906	27.5
45–64	8077	32.2
65+	6117	24.3
Gender		
Male	11,340	45.2
Female	13,773	54.8
Race/ethnicity		
White	20,972	83.5
Non-white	4141	16.5
Education Level		
Less than secondary school graduation	5338	21.3
Secondary school graduation	4022	16.0
Some post-secondary graduation	1655	6.6
Post-secondary graduation	14,098	56.1
Marital status		
Married/common law	12,637	50.3
Divorced/widowed/separated	5144	20.5
Single	7332	29.2
Household income		
Less than $20,000	1736	6.9
$20,000–$39,999	4410	17.6
$40,000–$59,999	5289	21.1
$60,000–$79,999	4190	16.6
$80,000+	9488	37.8
Immigration status		
Yes	4245	16.9
No	20,868	83.1
Type of smoker		
Daily smoker	4347	17.3
Occasional smoker	1200	4.8
Non-smoker	19,566	77.9
Type of drinker		
Regular drinker	14,210	56.5
Occasional drinker	5039	20.1
Non-drinker	5864	23.4
Have chronic conditions		
Yes	16,426	65.4
No	8687	34.6
Perceived health		
Excellent	4915	19.6
Very good	9443	37.6
Good	7437	29.6
Fair	2536	10.1
Poor	782	3.1

### Correlations of the studied variables

Table 2 presents zero-order correlations and descriptive statistics among variables included in the SEM analysis. None of the variable was severely skewed or kurtotic. Childhood maltreatment, social support and positive coping strategies were significantly correlated with MDE, GAD and suicide ideation. The full measurement model included two latent constructs, three observed variables of childhood maltreatment and five observed variables of social support. CFA results showed the measurement model provided adequate fit: CFI = 0.986; RMSEA = 0.055 (90% CI: 0.052, 0.057). The diagnostic plot suggested no difference between the missing and observed values (Fig. S1). All factor loadings for the indicators on the two latent variables were significant (*p* < 0.001), the loadings ranged from 0.45 to 0.79 for maltreatment and 0.77 to 0.89 for social support.

**Table 2:** Correlations among observed variables, means, and standard deviation

	1	2	3	4	5	6	7	8	9	10	11	12
1-IPV	–											
2-Physical abuse	0.51*	–										
3-Sexual abuse	0.29*	0.35*	–									
4-Positive coping skills	−0.06*	−0.07*	−0.08*	–								
5-Attachment	− 0.07*	− 0.08*	− 0.05*	0.25*	–							
6-Guidance	−0.07*	− 0.08*	− 0.06*	0.24*	0.79*	–						
7-Reliable alliance	−0.08*	− 0.09*	− 0.06*	0.24*	0.73*	0.75*	–					
8-Social integration	−0.09*	− 0.11*	− 0.08*	0.29*	0.70*	0.67*	0.66*	–				
9-Reassurance of worth	−0.06*	− 0.06*	− 0.05*	0.32*	0.67*	0.65*	0.62*	0.68*	–			
10-MDE	0.10*	0.14*	0.14*	−0.23*	−0.12*	−0.12*	− 0.13*	−0.15*	− 0.12*	–		
11-GAD	0.09*	0.12*	0.13*	−0.20*	−0.11*	− 0.10*	−0.11*	− 0.14*	−0.11*	0.35*	–	
12-Suicide ideation	0.11*	0.15*	0.12*	−0.16*	−0.12*	− 0.13*	−0.13*	− 0.14*	−0.11*	0.31*	0.21*	–
Mean (SD)	1.33 (0.94)	2.35 (1.24)	7.44 (1.62)	7.20 (1.02)	7.26 (1.03)	7.34 (0.93)	6.94 (1.12)	6.94 (1.05)	1.95 (0.22)	–	–	–
Missing data	9.31%	9.86%	9.65%	0.38%	0.80%	0.66%	0.49%	1.31%	2.55%	0.63%	0.90%	0.29%

*IPV* Exposure to intimate partner violence, *MDE* Major depressive episode, *GAD* Generalized anxiety disorder, *SD* Standard deviation

*p < 0.001(2-tailed)

### Structural model findings

**Fig. 1:**
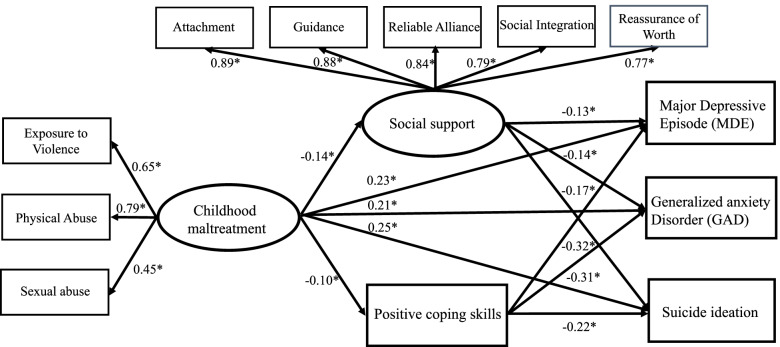
Measurement and structural model: Standardized factor loadings for final model of relationships between childhood maltreatment, positive coping skills, social support, and three psychaitrc disorders.*p < 0.05

Baron and Kenny’s 4-step criteria were met for the mediation analysis. Figure 1 shows the construction of the SEM with standardized path coefficients. Error covariance was added between social support and coping skills, and among three outcomes in the model to test hypotheses about shared sources of variability and to improve the model fit [53]. Results showed that direct, indirect and total effects were all significant. Childhood maltreatment was negatively associated with social support (β = − 0.138, *p* < 0.001) and positive coping skills (β = − 0.104, *p* < 0.001) and was positively associated with MDE (β = 0.227, *p* < 0.001), GAD (β = 0.206, *p* < 0.001) and suicide ideation (β = 0.249, *p* < 0.001). Social support was negatively associated with MDE (β = − 0.129, *p* < 0.001), GAD (β = − 0.140, *p* < 0.001) and suicide ideation (β = − 0.171, *p* < 0.001). In addition, positive coping strategy was negatively associated with MDE (β = − 0.317, *p* < 0.001), GAD (β = − 0.309, *p* < 0.001) and suicide ideation (β = − 0.218, *p* < 0.001). The SEM model provided a good fit for the data, the fit indices for the model were CFI = 0.983 and RMSEA = 0.056 (90% CI: 0.052, 0.058). Table 3 presents the results of direct, indirect, and total effects in the examined structural model. In terms of sensitivity analysis we also estimated the model without terror terms to further examine the robustness of these findings and the results remain similar (Table S1).

**Table 3:** Standardized direct, indirect effects and total effects for all hypothesized pathways

Mediating variable (M)	Effect of childhood abuse on M (a)	Effect of M on outcomes (b)	Indirect effect(a*b)	Direct effect (c’)	Total effect
**Outcome: Major depressive episode**					
Social support	−0.138**	−0.129**	0.018**	0.227**	0.278**
Positive coping skills	−0.104**	−0.317**	0.033**		
**Outcome: Generalized anxiety disorder**					
Social support	−0.138**	− 0.140**	0.019**	0.206**	0.257**
Positive coping skills	−0.104**	−0.309**	0.032**		
**Outcome: Suicide ideation**					
Social support	−0.138**	−0.171**	0.024**	0.249**	0.296**
Positive coping skills	−0.104**	−0.218**	0.023**		

**p < 0.01(2-tailed)

### Testing the indirect effects of social support and positive coping skills

The indirect effects of maltreatment on MDE through social support (B = 0.030, 95% CI: 0.024, 0.036), and positive coping skills (B = 0.055, 95% CI: 0.048, 0.063) were both statistically significant, respectively. The indirect effects of maltreatment on GAD via social support (B = 0.032, 95% CI: 0.026, 0.039), and positive coping skills (B = 0.054, 95% CI: 0.046, 0.062) were also statistically significant. Similarly, significant associations between maltreatment and suicide ideation were mediated by social support (B = 0.039, 95% CI: 0.033, 0.046), and positive coping skills (B = 0.038, 95% CI: 0.032, 0.044). By and large, the indirect effect of social support was weaker than positive coping skills. Taken together, we also found significant total indirect effects of social support and positive coping skills in the relationship between childhood maltreatment and MDE (β = 0.051, *p* < 0.001), GAD (β = 0.051, *p* < 0.001), and suicide ideation (β = 0.047, *p* < 0.001). Finally, our findings indicated the total effects of childhood maltreatment on MDE (β = 0.278, *p* < 0.001), GAD (β = 0.257, *p* < 0.001) and suicide ideation (β = 0.296, *p* < 0.001) through social support and coping skills were all significant (see Tables 3 and 4). These findings show that both social support and positive coping skills partially mediated the relationship between maltreatment and mental health outcomes. The total effects of childhood maltreatment on MDE, GAD and suicide ideation were mediated by both social support and coping skills in the order of 18.3, 19.8 and 15.9% respectively. The separate mediation proportions for positive coping skills on the association between childhood maltreatment and MDE, GAD and suicide ideation were respectively 11.9, 12.5 and 7.8%. For social support, the mediation proportions for the same disorders were 6.5, 7.4, and 8.1%, respectively.

**Table 4:** Summary of indirect effects among child abuse, social support, positive coping skills, major depressive episode, generalized anxiety disorder and suicide ideation

Path	Estimate	*p*-value	95% Bias-corrected CI	
			Lower	Upper
Child abuse→ Social support→ MDE	0.030	P < 0.01	0.024	0.036
Child abuse→ Social support→ GAD	0.032	P < 0.01	0.026	0.039
Child abuse→ Social support→ Suicide ideation	0.039	P < 0.01	0.033	0.046
Child abuse→ Positive coping skills→ MDE	0.055	P < 0.01	0.048	0.063
Child abuse→ Positive coping skills→ GAD	0.054	P < 0.01	0.046	0.062
Child abuse→ Positive coping skills→ Suicide ideation	0.038	P < 0.01	0.032	0.044

*MDE* major depressive episode, *GAD* generalized anxiety disorder, *CI* confidence interval

## Discussion

Our prevalence estimates of childhood maltreatment are comparable to a recent meta-analysis of 37 studies with a total of 253,719 participants [54]. By analyzing data from a large-scale national epidemiological survey the present study identified the buffering effects of personal resources (social support and positive coping strategies) in the associations between childhood maltreatment and MDE, GAD and suicide ideation, and compared the effects of these two mediators in those associations. Consistent with our hypotheses: 1) Both internal (coping skills) and external (social support) personal resources in later life were associated with childhood maltreatment and mental disorders. Coping strategies and social support had both independent and combined indirect effects on these studied mental health problems; 2) Coping strategies had a stronger impact on mental health problems compared to social support. The results of the current study are in line with previous evidence on the association between childhood maltreatment and a higher risk of mental disorders [55, 56] but extend those previous findings [56, 57]. The relationship between childhood maltreatment and the development of mental health problems is firmly established, however, the mediators of this link are less well understood. With coping as an internal resource and social support as an external resource, the current study expands the literature on personal resources mediating the associations between childhood maltreatment and subsequent mental health problems. The findings of the research not only illustrate the importance of personal resources in terms of reducing the negative consequences of childhood maltreatment on psychopathology but also suggest intervention and prevention strategies focusing on promoting personal resources to prevent the occurrence of mental health problems or ameliorate their severity among those with a history of maltreatment.

Both social support and coping strategies mediate the associations between childhood maltreatment and mental disorders. There is a direct effect of social support on mental health by providing emotional, instrumental, or informational assistance from a network of people drawn from family, friends and community [57]. Higher levels of social support have been linked with a lower risk of mental disorders [58]. We also found that social support may serve as an indirect path inhibiting the negative consequences of childhood maltreatment onto mental disorders. These findings support the stress-buffering model of social support which postulates that the presence of a social support system helps buffer, or shield, an individual from the negative impact of stressful events [59]. Our study is consistent with the existing literature highlighting the mediating role of social support in the relation between childhood maltreatment and anxiety as well as depression in adulthood [32, 34]. Consistent with our findings, Zheng et al., found in a sample of childhood trauma-exposed people that social support mediated the relationship between childhood abuse and suicidal ideation [60]. It is suggested that those with a history of childhood maltreatment may have limited access to the necessary supportive networks or resources to buffer these adverse effects, thus resulting in a higher frequency of psychiatric problems [61, 62].

Our study also showed significant indirect paths linking childhood maltreatment to mental disorders through positive coping strategies. Positive coping strategies in our study were associated with lower levels of major depressive episode, generalized anxiety disorder and suicide ideation. As Folkman notes that positive coping strategies can help individuals manage, reduce or tolerate demands originating from a stressful transaction [63]. The findings from our study are congruent with previous evidence about the mediation effect of positive coping strategies on the association between child maltreatment and depressive symptoms in adulthood [64]. In a similar vein, Calvete et al. in their study of female victims of intimate partner violence found that positive coping strategies mediated the impact of psychological abuse on mental health problems [65]. This evidence suggests that individuals exposed to childhood maltreatment may not have had the opportunity to learn to adequately cope with stressful events, which in turn increases the risk of anxiety and depression [66]. Further to this point, the current study is consistent with the hypothesis that trauma exposure influences the acquisition of an appropriate repertoire of coping skills, which, in turn, influences the development of psychopathology.

The research literature shows that positive coping strategies are strongly associated with a lower risk of major depressive episode, generalized anxiety and suicide ideation, whereas social support has a relatively weaker role in dampening the association between maltreatment and mental disorders [67, 68]. We found the same. This can be explained by the nature of personal resources, with coping strategies being internal and social support being external resources. It is not surprising that people tend to seek social support when they experience negative events, but it is confined and determined by the external environment whether they are able to obtain that support. The experience of maltreatment may prevent the disclosures of traumatic feelings/events for fear of stigmatization [69]. By contrast, coping strategies rely on one’s own conscious effort to balance internal and external demands, which is more reliable than depending on one’s ambient environment. However, although coping skills have a stronger impact on this association, it doesn’t mean that social support is of less importance. Increased levels of social support can help towards building significant resilience and promoting better coping strategies, and thus lessening the adverse mental health problems [70]. In addition, individuals who experience childhood maltreatment may experience considerable social rejection and exclusion, thus social support can play in protecting against such adverse mental health outcomes among maltreated people [71]. This study has several strengths: (1) the study data were from a large high-quality national health survey which provides robust and stable results and the study findings are generalizable to the Canadian population; (2) standardized instruments with structured interviews were used for the outcome assessment of MDE and GAD in the study; and (3) the use of Structural Equation Modeling and multiple mediation analysis provided the avenue to test the complex relationships among the studied variables. However, despite its many strengths this study has several limitations. First, the data for this study was cross-sectional. Causal interpretations of such research findings should be limited. As Baron and Kenny [43] note, meditation effects can only be examined when the temporal sequence association can be assumed which is the case between the experience of maltreatment in childhood and the later development of psychiatric disorders in adulthood. Although there is conceptual framework and empirical evidence supporting that childhood maltreatment leads to mental health problems through reduced social support and coping skills, it is also possible to postulate an alternate trajectory from childhood maltreatment through mental health problems to social support and coping skills deficits. However, based on the strong theoretical framework, we explored the more likely hypothesized model in the present study. Obviously, future longitudinal research is needed to further investigate these causal pathways and meditating effects. Second, measures of a child’s history of abuse were made through retrospective self-report. In addition, the CEVQ has yet to be validated in an adult population. They may be subject to recall bias as well as measurement error thus influencing the accuracy of the measurement of childhood maltreatment. Third, although the measurement on childhood maltreatment has an acceptable overall Cronbach’s α of 0.79, the factor loadings of sexual abuse was 0.45. Future research with improved reliability of the assessment of sexual abuse is needed. Fourth, it should be noted that even though the measurement of coping strategies has been used in the previous studies, it has not been validated and the psychometric properties needs to be further tested. Fifth, we conducted an unadjusted SEM without controlling potential confounders and covariates to eliminate confounding. However, Morgan argued that the potential confounding can be minimized by correlating error covariance of mediators and outcomes, which we did between the two mediators and the three outcomes studied in this analysis [72].

This present study analyzes the resilient mechanisms of personal resources in the association between childhood maltreatment and the specific mental disorders of major depressive episode, generalized anxiety disorder, and suicide ideation. Compared to external resources (social support), internal resources (positive coping strategies) played more important roles in these associations. The findings of the research provide a clear direction for prevention efforts aimed at to reducing the negative consequences of childhood maltreatment on the development of mental disorders.

## Supplementary Information

**Additional file 1: Figure S1**. Diagnostic plots for the mutiple imputation.

**Additional file 2: Table S1**. Sensitivity analysis on the standardized direct, indirect effects and total effects.

## Data Availability

The data that support the findings of this study are from the Public Use Microdata File (PUMF) of the Canadian Community Health Survey - Mental Health, Statistics Canada survey #5015. Access to the data is available to bona fide researchers through institutions participating in Statistics Canada Data Liberation Initiative (DLI) including university libraries throughout Canada - see https://www.statcan.gc.ca/eng/dli/dli. Access can also be arranged directly through DLI enquiries at: statcan.maddli-damidd.statcan@canada.ca.

## Abbreviations

CAPI: Computer assisted personal interviewing
CATI: Computer assisted telephone interviewing
CCHS-MH 2012: 2012 Canadian Community Health Survey-Mental Health
CEVQ: Childhood Experiences of Violence Questionnaire
CFA: Confirmatory factor analyses
CFI: Comparative Fit Index
CIs: Confidence intervals
DSM-IV: Diagnostics and Statistics Manual of Mental Disorder-4th edition
GAD: Generalized anxiety disorder
ICD-10: International Statistical Classification of Diseases and Related Health Problems-10th revision
MDE: Major depressive episode
MP: Mediation proportion
PUMF: Public Use Microdata file
RCMP: Royal Canadian Mounted Police
RMSEA: Sample root mean square error of approximation
SEM: Structural Equation Modeling
SPS-10: Social Provisions Scale-10 item
WHO-CIDI: World Health Organization version of Composite International Diagnostic Interview
WLSMV: Weighted least squares mean and variance adjusted test statistic
