# Supplementary figures and images for: The relationship between childhood maltreatment and mental health problems: coping strategies and social support act as mediators
Source: BMC Psychiatry. 2022 May 27;22:359. doi: 10.1186/s12888-022-04001-2 (PMC9137127; doi:10.1186/s12888-022-04001-2)

| (A) Physical abuse  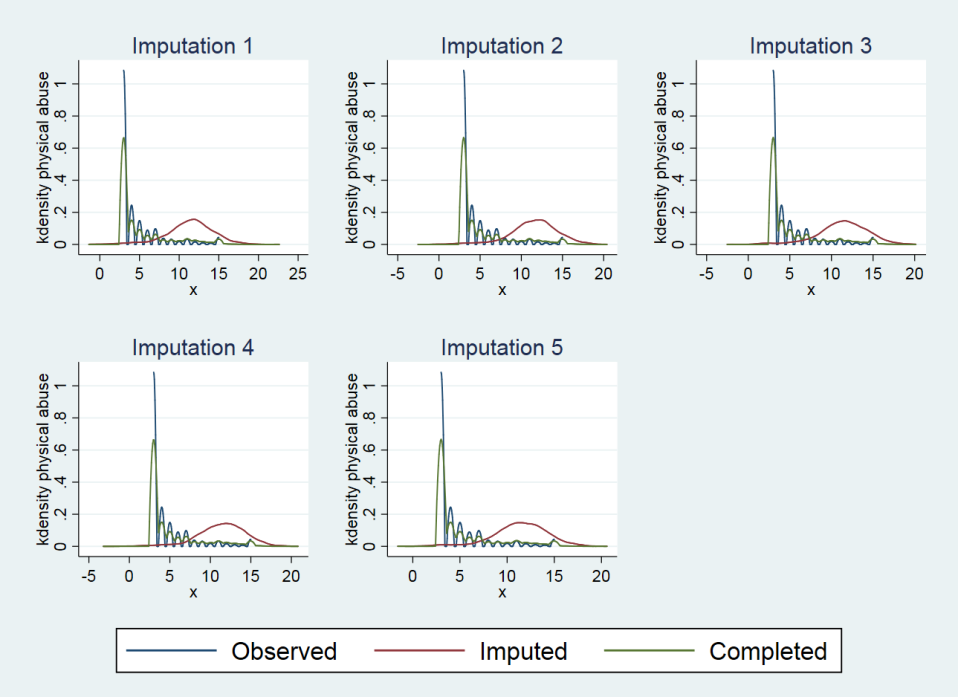 |
| --- |

(B) Sexual abuse


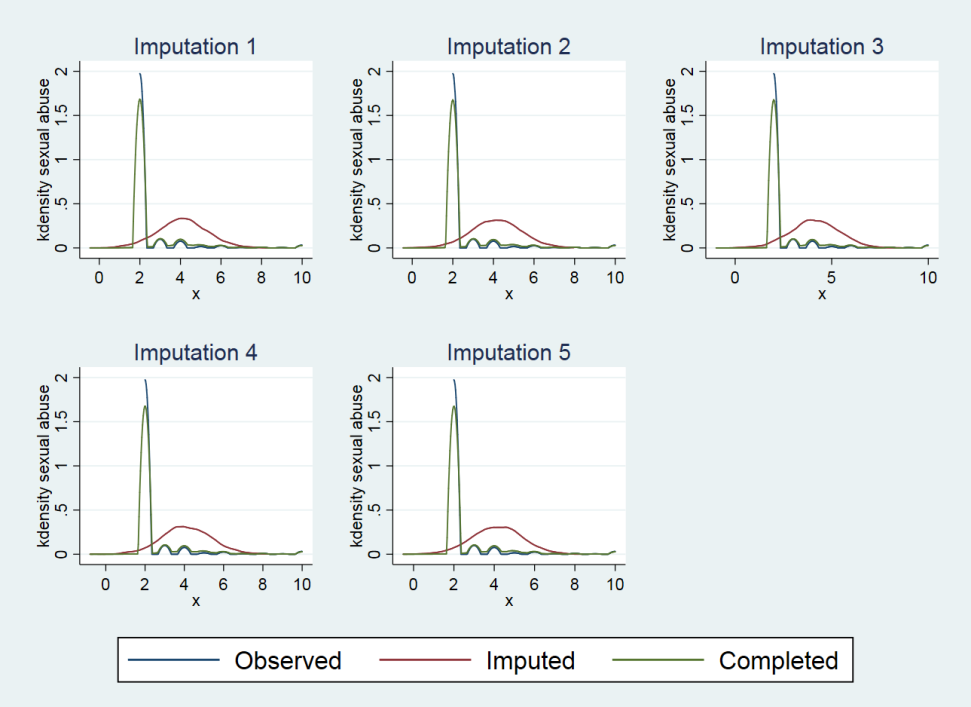


(C) Exposure to intimate partner violence


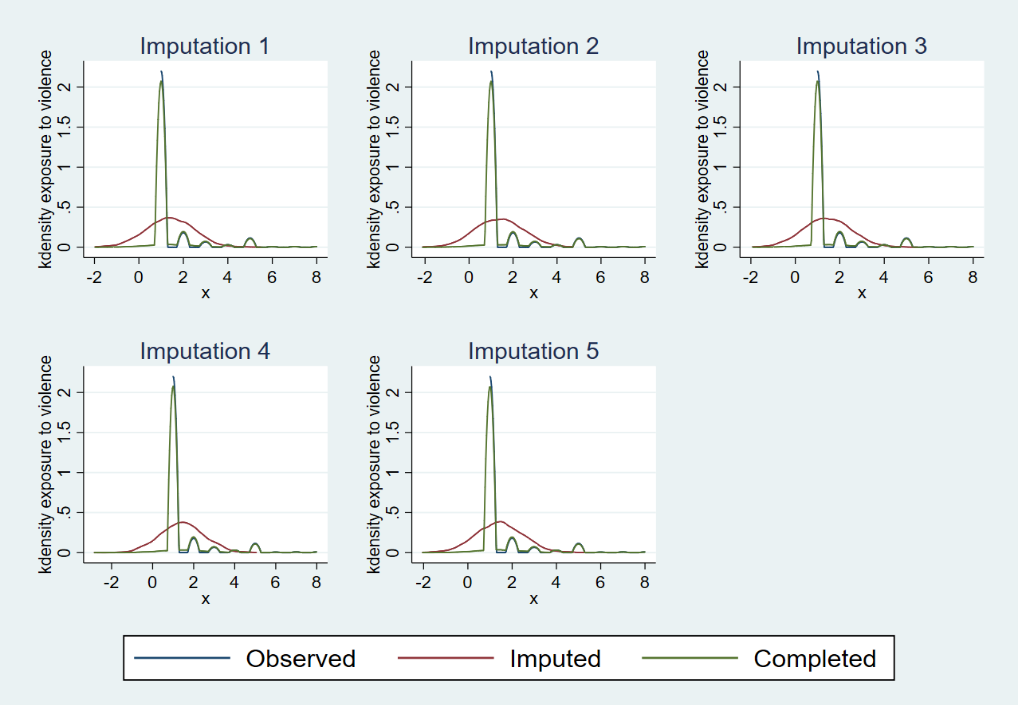

Supplement: Supplementary file 1 — Additional file 1: Figure S1. Diagnostic plots for the mutiple imputation. [file 12888_2022_4001_MOESM1_ESM.docx]
