# Supplementary material for: The relationship between childhood maltreatment and mental health problems: coping strategies and social support act as mediators
Source: BMC Psychiatry. 2022 May 27;22:359. doi: 10.1186/s12888-022-04001-2 (PMC9137127; doi:10.1186/s12888-022-04001-2)
Supplement: Supplementary file 2 — Additional file 2: Table S1. Sensitivity analysis on the standardized direct, indirect effects and total effects. [file 12888_2022_4001_MOESM2_ESM.docx]

| **Table S1.** Sensitivity analysis on the standardized direct, indirect effects and total effects. | | | | | |
| --- | --- | --- | --- | --- | --- |
| **Mediating variable (M)** | Effect of childhood abuse on M (a) | Effect of M on outcomes (b) | Indirect effect(a*b) | Direct effect (c’) | Total effect |
| **Outcome: Major depressive episode** |  |  |  |  |  |
| Social support | -0.207** | -0.139** | 0.029** | 0.427** | 0.512** |
| Positive coping skills | -0.286** | -0.196** | 0.056** |  |  |
| **Outcome: Generalized anxiety disorder** |  |  |  |  |  |
| Social support | -0.207** | -0.153** | 0.032** | 0.368** | 0.455** |
| Positive coping skills | -0.286** | -0.191** | 0.055** |  |  |
| **Outcome: Suicide ideation** |  |  |  |  |  |
| Social support | -0.207** | -0.187** | 0.039** | 0.447** | 0.526** |
| Positive coping skills | -0.286** | -0.139** | 0.040** |  |  |

*p<0.01(2-tailed).
